# Biosynthesis of Silver Nanoparticles from *Lotus sanguineus*: Photocatalytic, Antimicrobial, and Antibiofilm Activities with Molecular Docking Against Quorum-Sensing Proteins

**DOI:** 10.3390/biology15151261

**Published:** 2026-07-31

**Authors:** Suzan Şahin Doğan

**Affiliations:** Biology Department, KO Science Faculty, Karamanoglu Mehmetbey University, 70100 Karaman, Türkiye; sdogan@kmu.edu.tr

**Keywords:** green-synthesized silver nanoparticles, photocatalysis, antimicrobial and antibiofilm properties, molecular docking, *Lotus sanguineus*

## Abstract

Industrial effluents containing toxic dyes, pathogenic microorganisms, and other hazardous contaminants pose a serious threat to environmental and public health, creating an urgent need for multifunctional materials capable of addressing both environmental pollution and microbial contamination. Silver nanoparticles (AgNPs) have attracted considerable attention because of their broad-spectrum antimicrobial and photocatalytic properties. In this study, AgNPs were biosynthesized for the first time using *Lotus sanguineus*, an endemic plant rich in bioactive phytochemicals, through an environmentally friendly green synthesis approach. To comprehensively evaluate their multifunctional performance, the photocatalytic, antimicrobial, and antibiofilm activities of the biosynthesized AgNPs were investigated, while molecular docking analysis was performed to elucidate their interactions with quorum-sensing-related proteins. AgNPs were characterized using UV–Vis spectroscopy, FTIR, XRD, DLS, and SEM. Under sunlight, AgNPs showed degradation efficiencies of 60.6% for methylene blue and 29.5% for eriochrome black T, indicating higher photocatalytic activity toward the cationic dye. Antimicrobial evaluation demonstrated notable antimicrobial activities against *Escherichia coli*, *Bacillus cereus*, *Pseudomonas aeruginosa*, and *Staphylococcus aureus*, with minimum inhibitory concentration (MIC) values ranging from 31.25 to 62.5 μg/mL and minimum bactericidal concentration (MBC) values between 125 and 250 μg/mL. Additionally, AgNPs exhibited a maximum inhibition of 94.5% at 62.5 μg/mL against *Pseudomonas aeruginosa*. Furthermore, molecular docking results revealed favorable binding affinities between the ligand and quorum-sensing transcriptional activator (LasR), N-terminal domain of anthranilate-CoA ligase (PqsA), *P. aeruginosa* aminopeptidase (PaAP), and peptidoglycan N-acetylglucosamine deacetylase (Pgd) proteins. Overall, the results highlighted the potential of AgNPs for pharmaceutical applications, prevention of bacterial surface colonization, and environmental remediation.

## Simple Summary

Silver nanoparticles have attracted considerable attention because of their wide range of environmental and biomedical applications. In this study, the leaf extract of the endemic plant *Lotus sanguineus* was used as a natural and environmentally friendly reducing agent to produce silver nanoparticles. The synthesized nanoparticles were evaluated for their ability to degrade dye pollutants and inhibit the growth and biofilm formation of bacteria. Computer-based analyses were also performed to investigate their potential interactions with bacterial proteins. The results demonstrated promising photocatalytic, antibacterial, and antibiofilm properties, highlighting the potential of *Lotus sanguineus* for the sustainable production of silver nanoparticles for environmental and biomedical applications.

## 1. Introduction

Recently, natural product-mediated metallic nanoparticles have gained significant attention in biological, environmental, and pharmaceutical areas because of their numerous benefits over other methods, such as an eco-friendly synthesis process and low cost [1,2,3,4]. Natural products are valuable sources of vitamins, minerals, flavonoids, carotenoids, and alkaloids, which are used as capping and reducing agents in nanomaterial synthesis [5]. Thus, it has been stated that the biochemical properties of natural products can contribute to the biological, chemical, and catalytic characteristics of the synthesized nanoparticle [6,7,8].

Metal nanoparticles (such as gold, silver, and zinc) and natural polymers (e.g., chitosan) have been recognized for their roles as catalysts and sensors and in environmental remediation, drug delivery systems, and various medical applications, including anticancer, antimicrobial, and antibiofilm activities [9,10,11,12,13]. With increasing industrialization, wastewater pollution has become an important problem worldwide. Dyes used in industries such as textiles and paint were recognized as one of the important sources of water pollution, accounting for approximately 17–20% [7,14]. Synthetic organic dyes, which include methylene blue, congo red, saffranine, eosin Y, bromophenol blue, malachite green, and phenol red, were widely used in the dye industry [15]. Most textile dyes are known to be water-soluble and to cause environmental pollution, including eutrophication, reduced oxygen levels, the distribution of carcinogenic agents, and untreated dye effluents containing heavy metals [16]. Textile dyes produce large volumes of colorful effluents, which are a major source of environmental pollution and are difficult to break down using conventional techniques [17]. Nowadays, metal/metal oxide-based nanomaterials, such as silver, are attracting greater attention for the oxidation of hazardous compounds in nanobiotechnology [18,19]. Nanomaterial-based photocatalysts tend to improve the removal of dye contaminants at low concentrations because they have a higher surface area-to-mass ratio.

Among metal nanoparticles, silver nanoparticles exhibit strong absorption in the visible and sunlight regions, which has attracted significant interest for photocatalytic applications; moreover, environmentally friendly, cost-effective AgNPs have been successfully employed for the removal of textile dye contaminants, benefiting from their high surface-to-volume ratio [9,20,21]. In addition to their photocatalytic activity, nanoparticles have been reported to effectively prevent bacterial growth on membranes, a phenomenon known as membrane fouling. For example, it was reported that ZnFeCe nanoparticles were incorporated into membranes used for textile wastewater separation, thereby enhancing their antibacterial, photocatalytic, and separation performance [22,23].

Furthermore, AgNPs have received significant attention due to their strong biological activities, including antioxidant, anticancer, antimicrobial, and antibiofilm effects [4,24,25,26]. Biofilms, constructed by some microorganisms for self-protection, can cause significant problems, such as food contamination and the spread of bacterial infections [27]. There are several hypotheses that describe the antimicrobial, anti-inflammatory, and anticancer activities of AgNPs: disruption of membrane permeability, diffusion of nanoparticles into cells through membrane pores to inactivate proteins and DNA, and the generation of reactive oxygen species (ROS) [28].

Molecular docking has emerged as a computational tool for investigating the pharmacological activity of molecules with potential drug-like properties. It is applied to predict interactions between molecules and therapeutic targets [29]. Numerous studies have employed molecular docking approaches to explore the antimicrobial interactions of various chemicals, including zinc oxide nanoparticles, silver nanoparticles, Schiff bases, and natural compounds [30,31,32]. Molecular docking analysis in simulating interactions between silver nanoparticles and antibiofilm targets provides important in silico insights that support in vitro studies.

A number of plants, including *Hordeum vulgare*, *Plukenetia volubilis*, *Pterolobium hexapetalum*, *Valeriana jatamansi*, *Galphimia glauca*, and *Euphorbia sanguinea*, have been used for eco-friendly synthesis of silver nanoparticles [4,10,11,33,34]. Previous studies demonstrated that the genus *Dorycnium* contains a wide range of phytochemicals, including sulfur-based metabolites, isoflavonoids, flavonoids, hydroquinone, phenylbutanone glucosides, and various polyphenolic compounds. Therefore, it has been used in traditional medicine to treat various diseases [35,36,37,38]. Tosun et al. reported that *Lotus sanguineus* Vural D. D. Sokoloff (*Dorycnium sanguineum* Vural (syn.)) possessed high antioxidant activity and exhibited the highest total phenolic, phenolic acid, flavonoid, and proanthocyanidin contents [38]. Previous studies have highlighted the effectiveness of plant-derived metabolites in the production of bioactive AgNPs. Although the green synthesis of AgNPs using plant extracts has been tested with a wide variety of plants, it is noteworthy that each plant species possesses its own unique bioactive compounds [24].

The discharge of industrial effluents containing toxic and carcinogenic dyes, pathogenic bacteria, and other hazardous contaminants has become a major contributor to water pollution. These pollutants are predominantly released by the pharmaceutical, cosmetic, paint, and textile industries, posing significant risks to both the environment and public health [39]. The multifunctional materials’ ability to simultaneously degrade organic pollutants and inhibit microbes is highly desirable for wastewater treatment, membrane applications, and biomedical surface coating. Previous studies have demonstrated that AgNPs exhibit photocatalytic activity by generating reactive oxygen species that promote the degradation of organic pollutants under light irradiation [39,40]. Likewise, ROS generation has been widely recognized as one of the major contributors to the antimicrobial activity of metal nanoparticles by inducing oxidative stress, disrupting bacterial cell membranes, damaging proteins and nucleic acids, and ultimately leading to cell death [28]. Although the relative contribution of ROS depends on nanoparticle properties and experimental conditions, these findings highlight the multifunctional potential of AgNPs. Therefore, evaluating photocatalytic, antimicrobial, and antibiofilm activities together provides a comprehensive assessment of their applicability in environmental remediation and microbial control. Therefore, the synthesized nanoparticles were evaluated for their diverse applications. In this study, AgNPs were synthesized using the endemic plant *Lotus sanguineus* leaf extract due to its advantages over conventional methods, including the eco-friendly synthesis process, environmental sustainability, and cost-effectiveness. The photocatalytic activity of the synthesized silver nanoparticles was evaluated against both cationic and anionic dyes. In addition, molecular docking analyses were performed to investigate the potential interactions of the Ag_2_O structural model with quorum-sensing- and biofilm-associated proteins, including the quorum-sensing transcriptional regulator LasR, the N-terminal domain of anthranilate-CoA ligase (PqsA), *Pseudomonas aeruginosa* aminopeptidase (PaAP), and peptidoglycan N-acetylglucosamine deacetylase (Pgd), in order to provide mechanistic insight into their potential antibiofilm activity. This was complemented by in vitro evaluations of their antibiofilm and antimicrobial activities. To the best of our knowledge, this is the first report describing the green synthesis of AgNPs using the endemic plant *Lotus sanguineus*. Although numerous studies have investigated plant-mediated silver nanoparticles for antimicrobial applications, comprehensive studies integrating photocatalytic activity and antimicrobial and antibiofilm evaluations, together with molecular docking analysis, remain limited. Therefore, the present work not only expands current knowledge of the use of *Lotus sanguineus* as a natural reducing and stabilizing agent but also provides a more comprehensive assessment of the potential environmental and biomedical applications of the synthesized AgNPs.

## 2. Materials and Method

### 2.1. Collection and Authentication of Lotus sanguineus and Preparation of Leaf Extract

The leaves of *Lotus sanguineus* were collected from the Bucakkışla region of Karaman, Türkiye, on 2 September 2023. The collected plant samples were identified by Burak Surmen using several scientific publications [41,42,43]. *Lotus sanguineus* is an endemic species restricted to the Bucakkışla region of Karaman Province, Turkey, occurring in Çukur, Kurucabel, Aşağıakın, and Cerit at elevations of 350–900 m within an area of approximately 55 km^2^ [43]. Collected leaves were washed with distilled water and dried at room temperature for one week. Dried plant material was ground into a coarse powder, and 5 g of finely powdered leaves were mixed with 50 mL of distilled water and extracted for 2 h at 80 °C. The mixture was then filtered through Whatman No. 1 filter paper and stored at 4 °C for subsequent experiments.

### 2.2. Synthesis of Silver Nanoparticles by Lotus sanguineus

Ten milliliters of *Lotus sanguineus* extract was added dropwise to 30 mL of 1, 2, or 3 mM silver nitrate (AgNO_3_; Sigma-Aldrich, Saint Louis, MO, USA, Cat. No. 85228) solution under continuous stirring at 1000 rpm and 70 °C for 2 h. The initial pH of the reaction mixture was 6.7. During the reaction, a distinct color change from colorless to reddish-brown was observed following the addition of the pale-yellow plant extract, indicating the successful formation of silver nanoparticles. After 2 h, the reaction mixture was centrifuged at 15,000× *g* for 30 min. The resulting pellet was washed with distilled water and centrifuged at 15,000× *g* for 30 min. This washing and centrifugation procedure was repeated three times to remove residual impurities. Finally, the purified nanoparticles were air-dried at room temperature and stored at 4 °C in the dark until further characterization and subsequent experiments.

### 2.3. Characterization of Biosynthesized Nanoparticles

AgNPs were preliminarily characterized using UV–Vis spectroscopy during the synthesis process. The UV–Vis spectra of the plant extract and the synthesized AgNPs were recorded using a UV–Vis spectrophotometer (Thermo MultiSkan Go, Thermo Fisher Scientific, Vantaa, Finland) over a wavelength range of 300–800 nm at 30 min intervals. X-ray diffraction (XRD) analysis was performed to determine the crystalline phases of the synthesized AgNPs within a scanning range of 20–80°. The functional groups associated with the AgNPs were analyzed using Fourier transform infrared spectroscopy (FTIR; Vertex 70 ATR-FTIR spectrometer, Bruker, MS, USA) over a spectral range of 4000–400 cm^−1^. The hydrodynamic size distribution and zeta potential of the AgNPs were determined using a Zetasizer Nano ZS (Malvern Panalytical, Malvern, UK). Scanning electron microscopy (SEM) Hitachi SU-5000 (Hitachi High-Tech Corporation, Hitachinaka City, Japan) was employed to examine the morphology of the AgNPs, while their elemental composition was determined by energy-dispersive X-ray spectroscopy (EDX).

### 2.4. Antimicrobial Activity of Biosynthesized Silver Nanoparticles

Disc diffusion, minimum inhibitory concentration (MIC), and minimum bactericidal concentration (MBC) assays [44,45] were used to evaluate the antimicrobial activity of biosynthesized AgNPs against Gram-positive and Gram-negative bacteria. The smallest crystallite size among the tested concentrations (1, 2, and 3 mM) determined by XRD was selected for all subsequent biological assays.

#### 2.4.1. Disc Diffusion Method

Firstly, the antimicrobial activities of biosynthesized AgNPs were evaluated by a disc diffusion assay [44] against the following microorganisms: *Escherichia coli* ATCC 43895, *Staphylococcus aureus* ATCC 9144, *Bacillus cereus* NRRL B-3711, and *Pseudomonas aeruginosa* ATCC 27853. Overnight-grown bacterial cultures were adjusted to a 0.5 McFarland standard (1.5 × 10^8^ CFU/mL). Subsequently, 100 μL of each standardized bacterial suspension was evenly spread onto Mueller–Hinton agar (Merck; Cat. No. 1.05437) plates. All experiments were performed in triplicate. A total of 30 μL of AgNPs in the concentration range of 125–1000 μg/mL was applied to 6 mm sterile discs after vortexing. Sterile distilled water was used as the negative control, whereas tetracycline (TE, 30 μg/disc), penicillin (PE, 10 μg/disc), and gentamicin (CN, 10 μg/disc) served as positive controls. The plant extract was included as a reference sample. Inoculated plates were allowed to grow at 35 °C for 16–20 h, and the diameters of inhibition zones in all plates were recorded.

#### 2.4.2. MIC and MBC Assays

The MIC of AgNPs was determined against the tested bacteria. A total of 100 μL of the standardized bacterial suspension (final inoculum concentration: 10^5^ CFU mL^−1^) and 100 μL of AgNPs at concentrations ranging from 4 to 250 μg/mL were added to each well of a 96-well microplate and incubated at 35 °C for 16 h. Prior to each experiment, the AgNPs suspension was vortexed and sonicated. Each experiment was performed in triplicate. A total of 200 μL of bacterial culture without AgNPs was used as a positive control, while wells containing only AgNPs were used as the negative control. Culture medium alone served as the blank. After the MIC values for each tested bacterium were determined by visual observation and spectrophotometric measurement at 600 nm, 50 μL aliquots from wells showing no visible bacterial growth at the MIC and higher concentrations were spread onto Mueller–Hinton agar (MHA) plates. The plates were incubated at 35 °C for 16 h, and the minimum bactericidal concentration (MBC) was determined based on the presence or absence of bacterial colony growth.

### 2.5. Antibiofilm Activity

The antibiofilm effect of AgNPs was evaluated against *Pseudomonas aeruginosa* ATCC 27853 using the 96-well microtiter plate method. A total of 100 μL of AgNPs solution having different concentrations (3.9, 7.8, 15.6, 31.25, 62.5, 125, and 250 μg mL^−1^) and the same volume of overnight-grown bacterial culture adjusted to 10^5^ CFU mL^−1^ were inoculated into the sterile microplates and kept at 35 °C for 24 h. A total of 200 μL of bacterial culture without AgNPs was used as a positive control, while wells containing only AgNPs were used as the negative control. Culture medium (Mueller–Hinton broth) alone served as the blank. After incubation, the plates were washed three times with distilled water and left to dry. A total of 200 μL of 0.1% crystal violet (Merck; Cat. No. 1.15940) solution (*w*/*v*) was applied to stain the adherent biofilm on the plate for 15 min. The excess crystal violet dye was removed by washing the microplates three times with distilled water, and the adhered biofilm was then treated with 200 μL of absolute ethanol for 15 min. The plates’ optical density (OD) was measured at 595 nm with a UV–Vis spectrophotometer. Before statistical analysis, the absorbance values were corrected by subtracting the mean absorbance of the blank wells. One-way ANOVA with Tukey’s post hoc test was used to assess differences in biofilm formation among various AgNP concentrations using OriginPro 2026 SR1 V10.3.0.197. Biofilm inhibition (%) was calculated according to the following equation:

$$\left(\%\right)Inhibition=\frac{{OD}_{control}-{OD}_{sample}}{A_{control}}\times 100$$

### 2.6. Molecular Docking

In the present study, molecular docking analysis was performed on proteins involved in microbial pathogenesis and biofilm formation. The crystal structures of target proteins, including LasR (4NG2), PqsA (5OE5), PaAP (8AC7), and Pgd (4L1G), were retrieved from the RCSB Protein Data Bank (PDB) using the PDB IDs [46]. Also, the crystal structure of the Ag_2_O molecule was obtained from the Materials Project website as a simplified molecular model to investigate protein–nanoparticle interactions. The 2D structure of gentamycin was retrieved from PubChem and used as a reference drug. Discovery Studio V24.1.0.23298 [47] was used to remove heteroatoms, water molecules, and ligands from all crystal structures of target receptors, which were then saved in PDB format. The ligand was converted to PDB format and minimized using Open Babel 3.1.1 and Chimera 1.18 [48,49]. AutoDock Tools 1.5.7 was used to add polar hydrogen atoms and Kollman charges to the target molecules and to merge nonpolar hydrogen atoms [50]. The files were then saved in pdbqt format for docking analysis. AutoDock Tools 1.5.7 was used to perform molecular docking employing the Lamarckian Genetic Algorithm (LGA), with 10 docking runs and a maximum of 2,500,000 energy evaluations. The grid box was defined with dimensions of 90 × 90 × 90 grid points and a grid spacing of 0.375 Å. The docking protocol was validated by redocking the co-crystallized ligand (N-3-oxo-dodecanoyl-L-homoserine lactone) into the LasR binding pocket (PDB ID: 4NG2) using the same docking parameters. The crystallographic and predicted binding poses were compared using the RMSD/Superimpose function in AutoDock Tools 1.5.7. The docking log files (dlg) were examined to identify the best-docked conformation with the lowest binding free energy for the ligand. AutoDock Tools 1.5.7 and Discovery Studio V24.1.0.23298 were used to analyze docking results.

### 2.7. Photocatalytic Activity

Cationic and anionic dye solutions were used to investigate the photocatalytic activity of the AgNPs based on a slightly altered protocol implemented by Berkani et al. [51]. A total of 10 mL of 10 ppm methylene blue (MB) and eriochrome black T dye (EBT) solutions were prepared separately. Next, the dye solutions were stirred in the dark at room temperature for 20 min to reach adsorption–desorption equilibrium after adding 1 mg of AgNPs to both MB (pH 5.58) and EBT (pH 6.22) solutions. The mixtures were continuously stirred at 200 rpm. MB and EBT solutions without AgNPs, under both sunlight and dark conditions, were used as controls. Afterward, the control and catalyst-added dye solutions were stirred under sunlight for 3 h. A total of 300 μL of the solution was subjected to centrifugation at 10,000× *g* for 10 min at 30 min intervals, and each separated supernatant was measured by UV–Vis between 300 and 800 nm. Also, the absorbance spectra of MB and EBT were noted in 30 min intervals over 3 h using a UV–Vis spectrophotometer. Photocatalytic degradation experiments. The photocatalytic experiments were performed under natural sunlight on 20 July between 12:00 and 15:00. During the experiments, the solar irradiance was approximately 3.6–4.0 kWh/m^2^/day. The decolorization percentage was computed using the following equation:

(1) $$Decolorization\left(\%\right)=\frac{A_{0}-A_{t}}{A_{0}}\times 100$$

where A_0_ represents the initial absorbance of the dye, and A_t_ denotes the absorbance of the dye at time *t* (min). Furthermore, the pseudo-first-order reaction kinetics were evaluated using the following equation:

(2) $$\ln\left(\frac{C_{0}}{C_{t}}\right)=-k_{app}t\;\;(Langmuir-Hinshelwood\;model)$$

The apparent rate constant (*k_app_*, min^−1^) was obtained from the slope of the linear regression plot of ln(C_0_/C_t_). In Equation (2), C_t_ represents the dye concentration at time *t*, whereas C_0_ corresponds to the initial dye concentration at the beginning of irradiation.

## 3. Results

**Figure 1:**
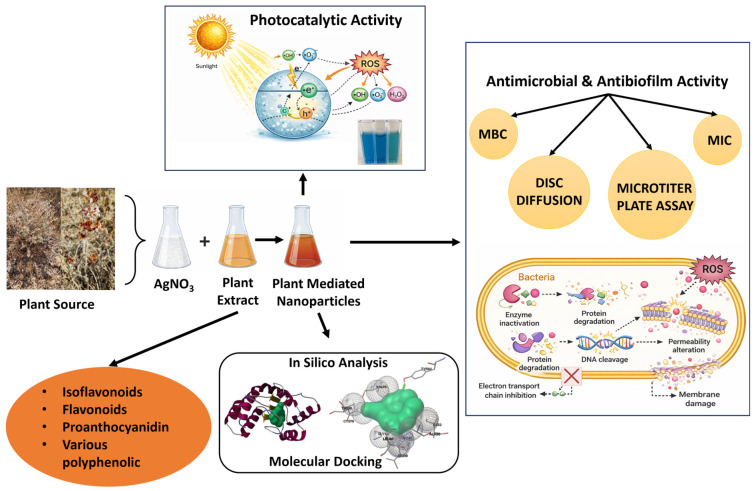
Diagrammatic representation of the synthesis of AgNPs and the applied bioassays.

A green protocol for producing AgNPs was demonstrated by reducing silver ions using sustainable resources, with plant extract serving as both a stabilizing and reducing agent, without the need for harmful or expensive chemicals. The photocatalytic, antimicrobial, and antibiofilm activities of AgNPs were investigated (Figure 1).

The formation of Ag/Ag_2_O nanoparticles through green synthesis is initiated by the reduction of Ag^+^ ions using bioactive constituents present in the *Lotus sanguineus* extract under aqueous conditions, eliminating the need for hazardous chemicals or organic solvents. The formation of Ag/Ag_2_O nanoparticles is proposed to occur through the reduction of Ag^+^ ions by phytochemicals present in the *Lotus sanguineus* extract, which also act as stabilizing agents during nanoparticle synthesis. The bioactive compounds facilitate the reduction of Ag^+^ ions, leading to the generation of nanoscale Ag^0^/AgOH clusters. Following the reduction of Ag^+^ ions, dissolved oxygen facilitates the partial oxidation of metallic silver, leading to the formation of AgOH/Ag^0^ clusters [52,53]. During drying, unstable silver hydroxide intermediates are converted to Ag_2_O, yielding Ag/Ag_2_O nanoparticles, as confirmed by XRD.

### 3.1. Characterization of Biosynthesized AgNPs

**Figure 2:**
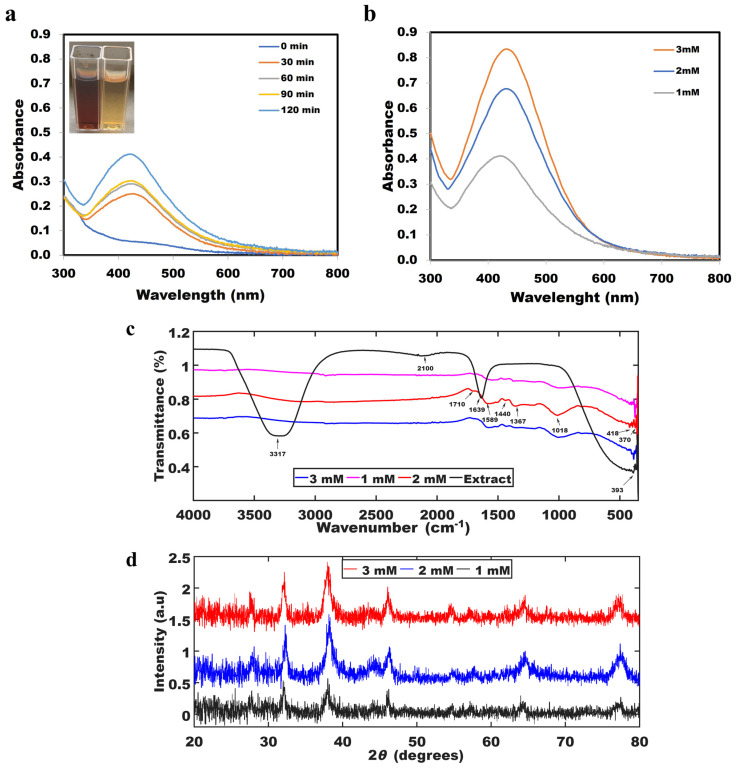

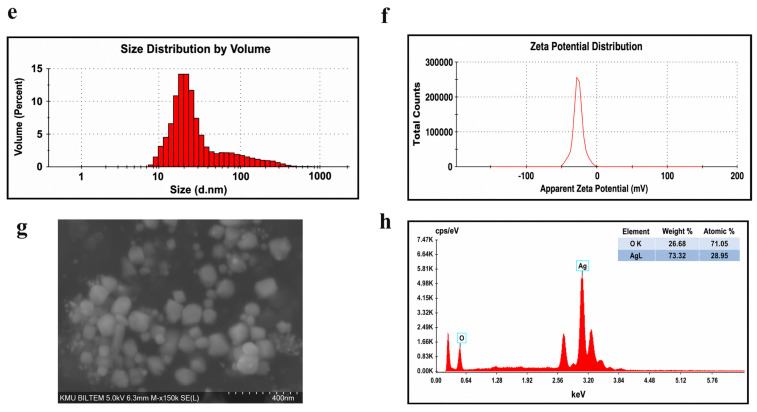
Characterization of the biosynthesized AgNPs. (**a**) Effect of reaction time on the synthesis of AgNPs (1 mM AgNO_3_), including the color change observed during nanoparticle formation and the plant extract. (**b**) Effect of AgNO_3_ concentration on AgNP synthesis. (**c**) FTIR spectrum of the biosynthesized AgNPs. (**d**) XRD pattern of the biosynthesized AgNPs. (**e**) Particle size distribution determined by dynamic light scattering (DLS). (**f**) Zeta potential of the biosynthesized AgNPs. (**g**) SEM image showing the morphology of the biosynthesized AgNPs. (**h**) EDX spectrum showing the elemental composition of the biosynthesized AgNPs.

First, the characterization of AgNPs was performed using UV–Vis spectroscopy over the wavelength range of 300–800 nm. UV–Vis spectroscopy is an efficient and widely used technique for nanoparticle characterization, owing to the excellent light-absorption properties of metal nanoparticles. In the current study, the absorption peak of the biosynthesized AgNPs was detected at 430–445 nm (Figure 2a). Increasing the AgNO_3_ concentration resulted in a gradual increase in absorbance intensity, while the SPR band shifted from 430 to 440 nm (Figure 2b).

The FTIR spectrum of the *Lotus sanguineus* extract exhibited a broad absorption band at 3317 cm^−1^**,** together with peaks at 2950, 2850, 2100, 1639, 1710, 1589, and 1018 cm^−1^**,** indicating the presence of hydroxyl, aliphatic C–H, carbonyl, amide, and C–O functional groups (Figure 2c). In addition, distinct absorption bands were observed at 1710, 1639, 1589, 1440, 1367, 1018, 418, and 370 cm^−1^**.** The bands at 370 and 418 cm^−1^ were assigned to metal–biomolecule complexes, confirming the interaction between silver species and plant-derived biomolecules during nanoparticle formation.

XRD analysis confirmed the formation of both Ag and Ag_2_O crystalline phases (Figure 2d). Diffraction peaks observed at 32.25°, 38.16°, 46.29°, 64.59°, and 77.38° corresponded to the (110), (111), (200), (222), and (311) crystal planes, respectively. The crystallite sizes calculated using the Debye–Scherrer equation were 27, 12, and 14 nm for nanoparticles synthesized with 1, 2, and 3 mM AgNO_3_, respectively. The average crystalline size of the AgNPs, as a function of the width of the (111) diffraction peak lines for 1-, 2-, and 3 mM concentrations, was calculated using the Debye-Scherrer formula. The crystallite sizes were found to be 27, 12, and 14 nm, with corresponding FWHM values of 0.315, 0.774, and 0.660, respectively. The average crystallite size was estimated using the Debye–Scherrer equation, D = Kλ/(βcosθ), where K = 0.89 is the shape factor, λ = 1.5406 Å is the wavelength of Cu Kα radiation, β is the full width at half maximum (FWHM) of the (111) diffraction peak expressed in radians, and θ is the corresponding Bragg angle.

The average size of the synthesized AgNPs was determined using Dynamic Light Scattering (DLS), as shown in Figure 2e. Although most nanoparticles were smaller than 100 nm, the synthesized nanoparticles had an average size of 104.7 nm ± 3.3 nm and a polydispersity index (PDI) of 0.285 ± 0.012 (Figure 2e). Additionally, the zeta potential of the biosynthesized AgNPs was measured at −23 mV, with a mobility of −1.806 (μm·cm/V·s) (Figure 2f).

SEM images of the AgNPs produced from the 2 mM AgNO_3_ solution revealed that most nanoparticles were spherical, although some were irregular in size and shape (Figure 2f,g). Additionally, EDX analysis confirmed the presence of elemental silver and oxygen (Figure 2h).

### 3.2. Antimicrobial and Antibiofilm Activity

**Figure 3:**
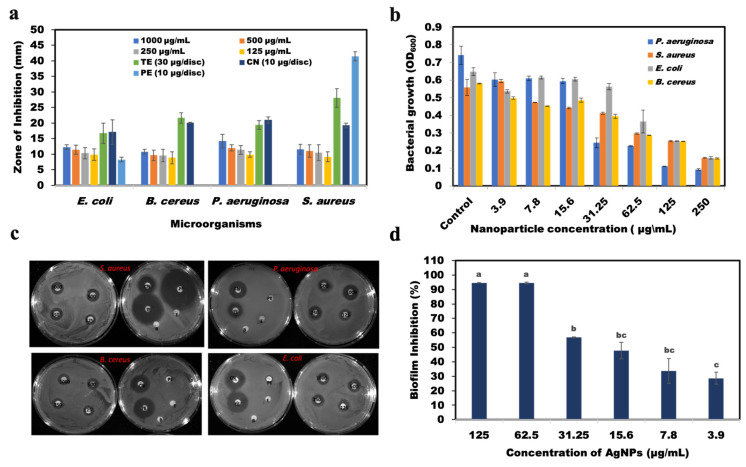
The antimicrobial and antibiofilm activity of AgNPs. (**a**) Zone of inhibition of AgNPs against *E. coli*, *B. cereus*, *P. aeruginosa*, and *S. aureus* (TE, tetracycline (30 μg/disc); CN, gentamicin (10 μg/disc); PE, penicillin (10 μg/disc)). (**b**) The effect of AgNPs on bacterial growth. (**c**) The images of the disc diffusion assay for silver nanoparticles (antibiotic discs used: tetracycline (TE, 30 μg/disc), penicillin (PE, 10 μg/disc), and gentamicin (CN, 10 μg/disc)). B represents the negative control, E represents the plant extract, and AgNPs were tested at concentrations ranging from 125 to 1000 μg/mL. (**d**) Inhibition percentage of AgNPs at different concentrations against *P. aeruginosa*. Values are means ± the standard error (n = 3). Groups marked with different letters above the error bars are significantly different, as determined by Tukey’s test at *p* < 0.05.

The inhibition zone diameters and minimum inhibitory concentration (MIC) values of AgNPs and standard antibiotics, including penicillin, tetracycline, and gentamicin, for each bacterial strain, as well as OD values of the tested microorganisms, were given in Table 1 and Figure 3a,b. Minimum bactericidal concentrations (MBC) and MIC values of AgNPs against *Escherichia coli* ATCC 43895, *Bacillus cereus*, *Pseudomonas aeruginosa* ATCC 27853, and *Staphylococcus aureus* ATCC 9144 were determined to be 125, 125, 125, and 250 (MBC) and 62.5, 62.5, 31.25, and 62.5 μg/mL (MIC), respectively, as shown in Table 1. The inhibition zones were 12.25, 10.80, 14.20, and 11.55 mm for *E. coli*, *B. cereus*, *P. aeruginosa*, and *S. aureus* (Figure 3a). According to the biofilm formation assay, AgNPs at different concentrations exhibited statistically significant (*p* < 0.05) reductions compared to the control (Figure 3c and Figure S1).

### 3.3. Molecular Docking

**Figure 4:**
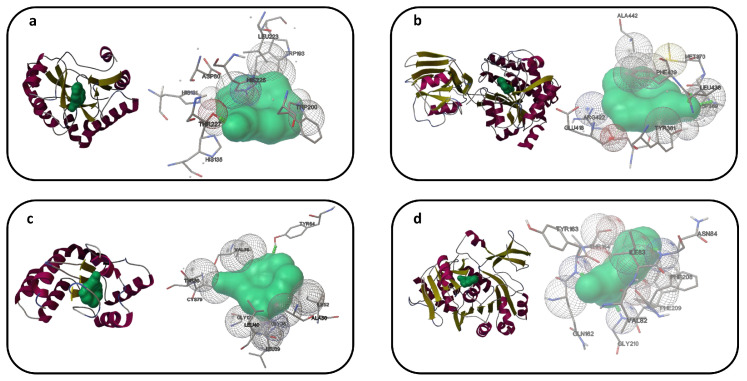
Receptor–ligand complex and interaction of common residues at the binding pocket. (**a**) Pgd, (**b**) PaAP, (**c**) LasR, (**d**) PqsA.

Molecular docking simulations in the present study revealed binding affinities between the ligand and the Pgd, PaAP, LasR, and PqsA proteins of −3.84, −3.67, −4.25, and −5.63 kcal/mol, respectively (Figure 4a–d, Table 2 and Table 3). For comparison, the reference ligand gentamicin exhibited docking scores of −3.29, −9.46, −3.16, and −2.23 kcal/mol against Pgd, PaAP, LasR, and PqsA, respectively. The docking protocol was validated by redocking the co-crystallized ligand (N-3-oxo-dodecanoyl-L-homoserine lactone) into the LasR binding pocket using the same docking parameters. The redocked pose yielded an RMSD value of 1.607 Å relative to the crystallographic ligand (Figure S2).

### 3.4. Photocatalytic Activity

**Figure 5:**
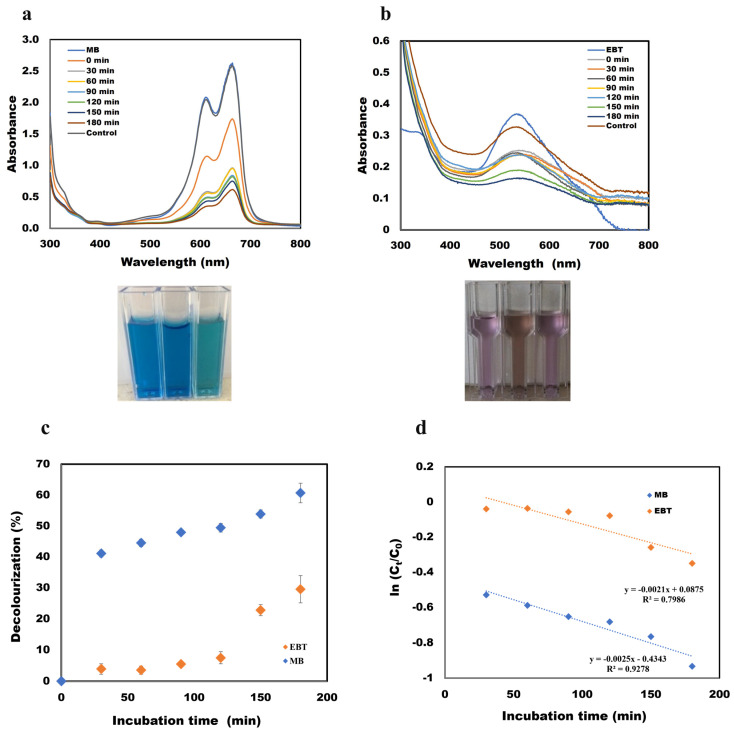
Photodegradation of MB and EBT dye solution treated by AgNPs. (**a**) UV–Vis spectra of MB at different time intervals (tubes (from left to right) showing MB without AgNPs under sunlight, MB without AgNPs in the dark, and MB with AgNPs under sunlight). (**b**) UV–Vis spectra of EBT at different time intervals (tubes (from left to right) showing EBT without AgNPs under sunlight, EBT with AgNPs under sunlight, and EBT without AgNPs in the dark). (**c**) Decolorization percentage of MB and EBT by AgNPs. (**d**) Pseudo-first-order kinetics of MB and EBT photodegradation by AgNPs. Values are means ± the standard error of the mean (SEM) (n = 2–3).

The photocatalytic activity of biosynthesized AgNPs was investigated against anionic and cationic dyes. The photodegradation of MB and EBT under sunlight was analyzed using UV–Vis spectroscopy, and changes in color intensity were visually observed. Firstly, the characteristic absorption peaks of MB and EBT were observed at 665 and 540 nm (Figure 5a,b), consistent with prior data [21]. The absorbance of the photocatalytic degradation of EBT and MB was recorded at 30 min intervals over 3 h. In the first 30 min, AgNPs rapidly degraded 30% of the MB. The results showed that AgNPs degraded 60.6% of the MB dye after 3 h of incubation under sunlight. Additionally, 29.5% of EBT was degraded at the same photocatalyst concentration and incubation time (Figure 5c). Moreover, the pseudo-first-order kinetic model showed a correlation (R^2^ = 0.92) with a corresponding rate constant (k_1_ = 0.0025 min^−1^) for MB, whereas R^2^ = 0.79 with a rate constant (k_1_ = 0.0021 min^−1^) for EBT (Figure 5d). The color changes in the MB and EBT were visually observed after 3 h of incubation with AgNPs under sunlight. On the other hand, the controls showed no visible color change with time (Figure 5a,b). After 3 h of sunlight exposure without AgNPs (control), decolorization of approximately 3% and 11% was observed for MB and EBT solutions, respectively (Figure 5a,b).

## 4. Discussion

### 4.1. Characterization of Biosynthesized AgNPs

The color change from a colorless silver nitrate solution to reddish-brown was considered a preliminary visual indication of the development of a surface plasmon resonance (SPR) band [54], suggesting the possible formation of AgNPs, which was later confirmed by spectroscopic characterization (Figure 2a). It has been reported that decreasing silver nanoparticle size causes a spectral shift toward the blue or red region, resulting in absorption maxima at shorter wavelengths, a state of aggregation, and higher energies [55]. Moreover, at lower AgNO_3_ concentrations, the SPR band became broader and weaker, implying the formation of silver nanoparticles with a broader particle size distribution [56]. According to the UV–Vis spectra, both the maximum absorption wavelength and the yield of the final product increased with increasing AgNO_3_ concentration (Figure 2b). The sharp and symmetrical SPR band at approximately 430 nm suggested the formation of non-aggregated nanoparticles [57,58]. As the AgNO_3_ concentration increased, the surface plasmon resonance (SPR) band shifted from 430 to 440 nm, which may be attributed to changes in nanoparticle size, aggregation state, and/or interparticle interactions. XRD analysis showed that the crystallite size decreased markedly from 27 nm at 1 mM AgNO_3_ to 12–14 nm at 2–3 mM AgNO_3_, while only a slight difference was observed between the 2 and 3 mM samples.

The broad peak at 3317 cm^−1^ may correspond to O–H stretching vibrations, while the peak at 2100 cm^−1^ could be attributed to alkyne (–C≡C–) stretching, indicating the presence of nitrile or alkyne-related phytoconstituents originating from plant metabolites [59]. The intense band at 1639 cm^−1^ was attributed to carboxylic C=O stretching, likely originating from polyphenolic compounds present in the plant extract [11,60]. Aliphatic C–H bending vibrations were indicated by the small absorption bands at 2850 and 2950 cm^−1^. Additionally, the bands at 1710 and 1589 cm^−1^ further support the presence of carboxylic and amide groups. Furthermore, the band at 1018 cm^−1^ was attributed to the C–O stretching vibrations of primary and secondary alcohol groups [61], indicating that plant-derived compounds contributed to nanoparticle stabilization. Compared with the plant extract, the FTIR spectrum of the synthesized AgNPs showed a markedly less intense broad band at 3317 cm^−1^, while the band at 2100 cm^−1^ disappeared. In contrast, new absorption bands appeared at 1710, 1639, 1589, 1440, 1367, 1018, 418, and 370 cm^−1^ (Figure 2c). The absorption peaks at 370 cm^−1^ and 418 cm^−1^ indicated the presence of metal–biomolecule complexes in the sample [62]. These findings demonstrate that flavonoids, phenolic acids, and protein-associated functional groups in the *Lotus sanguineus* extract serve dual roles as reducing and capping agents, forming a bioactive organic corona around AgNPs. Overall, FTIR results confirmed that phenolic and carbonyl-containing phytochemicals from *Lotus sanguineus* extract functioned as reducing and stabilizing agents during green synthesis, forming a biofunctional organic capping layer that may enhance antimicrobial activity.

The XRD results revealed the presence of both Ag2O and Ag nanoparticles in the sample. In Figure 2d, peaks represented at a 2θ of 32.25° (Ag_2_O), 38.16° (Ag/Ag_2_O), 46.29° (Ag), 64.59° (Ag), and 77.38° (Ag) correspond to the (110), (111), (200), (222), and (311) planes of the cubic crystalline structure of metallic silver and silver oxide (JCPDS file No. 5-2872). XRD patterns of the synthesized Ag/Ag2O were consistent with those reported in similar studies mediated by biomaterials [6,51,63].

The zeta potential of the biosynthesized AgNPs indicated good dispersion in liquids, and the negative zeta potential values confirmed repulsion between the nanoparticles, ensuring their stability [25]. A zeta potential value of −23 mV indicated moderate colloidal stability. According to previous studies, nanoparticle dispersions with absolute zeta potential values approaching or exceeding ±30 mV generally exhibit improved electrostatic stability, whereas lower absolute values may permit gradual aggregation during prolonged storage [64]. The AgNPs synthesized from the 2 mM AgNO_3_ solution exhibited the smallest crystalline size. It was observed that the size of the AgNPs increased with increasing AgNO_3_ concentration [20]. Conversely, AgNPs synthesized using a 2 mM AgNO_3_ solution exhibited smaller crystalline sizes compared to those synthesized with 1 mM and 3 mM AgNO_3_ solutions. The larger DLS size reflects the hydrodynamic diameter of nanoparticles in suspension, including the electrostatic ion layer surrounding the particles and possible agglomeration, whereas SEM provides the physical size of dry particles [65].

### 4.2. Antimicrobial and Antibiofilm Activity

First, the agar disc diffusion method was employed to evaluate the antibacterial properties of the AgNPs. The AgNPs displayed visible inhibition zones against the tested bacterial strains. The AgNPs exhibited significant bacteriostatic and bactericidal activity against the tested bacterial strains. The MIC and MBC results revealed concentration-dependent antimicrobial activity of AgNPs with strain-specific differences. In the disc diffusion assay, the zone of inhibition increased with increasing nanoparticle concentration from 125 to 1000 μg/mL for all tested strains. The largest inhibition zones were observed at 1000 μg/mL against *P. aeruginosa* (12.57 mm). OD values of the tested microorganisms exposed to AgNPs demonstrated that the nanoparticles effectively inhibited the growth of both Gram-negative and Gram-positive bacteria (Figure 3b,c). According to the biofilm formation assay, AgNPs at different concentrations showed statistically significant reductions (*p* < 0.05) compared to the control (Figure 3c). Moreover, the antibiofilm activity increased in a concentration-dependent manner. Inhibition values rose from 28.6% at 3.9 μg/mL concentration to 56.8% at 31.25 μg/mL, indicating progressive suppression of biofilm formation. A maximum inhibition of 94.5% was achieved at a 62.5 μg/mL concentration and remained unchanged at higher concentrations (125 μg/mL), suggesting a plateau effect. These results indicate strong anti-biofilm activity, with 62.5 μg/mL identified as the minimum concentration required for near-complete biofilm inhibition (Figure 3d).

In a previous study, silver nanoparticles synthesized from the fruit extract of *Mimusops elengi* showed the highest antibacterial activity against Gram-positive bacteria at 312.5 µg/mL and against Gram-negative bacteria at 1250 µg/mL. Additionally, MBCs were reported as 625 µg/mL for Gram-positive and 250 µg/mL for Gram-negative bacteria [66]. Moreover, Berkani et al. [51] reported that AgNPs produced by the strain S16 at a concentration of 18.200 μg/mL exhibited antimicrobial activity, with inhibition zones ranging from 8 to 12 mm, against *E. coli* ATCC 25922, *P. aeruginosa* ATCC 27853, *B. cereus*, and *S. aureus* ATCC 25923. In the same study, the lowest MIC (284.375 μg/mL) was observed against E. coli, while the highest (1137.5 μg/mL) was observed against *P. aeruginosa.* Wang et al. (2022) demonstrated that probiotic-derived AgNPs inhibited *E. coli* ATCC 43895 at an MIC of 3.12 μg/mL [67]. Similarly, Jaiswal and Mishra (2018) reported an MIC of 5 mg/mL for curcumin-silver nanoparticles against *S. aureus* ATCC 9144 [68]. In addition, it was reported that *Senna italica*-mediated AgNPs were shown to produce a zone of inhibition of 11 ± 1.2 mm against *S. Typhimurium* ATCC 14028 [69]. In another study, AgNPs synthesized using the extract of *G. lanceolarium* showed MIC values of 43.94 μg/mL, 68.6 μg/mL, and 44.02 μg/mL against *S. aureus*, *P. aeruginosa*, and *E. coli*, respectively [24]. In the present study, the lowest and highest MIC values were 31.25 and 62.5 µg/mL, respectively (Table 1). Additionally, the lowest MBC value (125 μg/mL) was observed against *Escherichia coli*, *Bacillus cereus*, and *Pseudomonas aeruginosa*, whereas the highest MBC value (250 μg/mL) was recorded against *Staphylococcus aureus*. (Table 1). These results demonstrated that AgNPs synthesized using *Lotus sanguineus* leaf extract possessed promising antimicrobial activity against both Gram-positive and Gram-negative microorganisms, even at relatively low concentrations. AgNPs showed slightly better antimicrobial performance (MIC and MBC) than reported in some published literature. This enhanced activity may be associated with bioactive metabolites from the *Lotus sanguineus* extract, which likely acted as reducing and stabilizing agents during nanoparticle synthesis and may have influenced the nanoparticles’ physicochemical properties, including size and surface characteristics. Several mechanisms have been proposed in the literature to explain the antimicrobial activity of AgNPs. These include the attachment of nanoparticles to the bacterial cell surface, resulting in membrane disruption and damage to intracellular components; the release of Ag^+^ ions that interact with sulfhydryl groups of proteins and enzymes; and the generation of reactive oxygen species (ROS), which induce oxidative stress and interfere with essential cellular functions [6,28]. However, these mechanisms were not directly investigated in the present study; therefore, they should be regarded as possible explanations based on previous reports rather than confirmed mechanisms underlying the observed antimicrobial activity.

*P. aeruginosa* was selected for the antibiofilm assay due to its strong biofilm-forming ability [70]. The ability of *P. aeruginosa* to form biofilms contributes significantly to its role in persistent infections and reduced antibiotic efficacy, often leading to prolonged healing times or inappropriate antibiotic use, which in turn promotes the development of microbial resistance [71]. The anti-biofilm activity of AgNPs was evaluated using a microtiter plate assay across a range of concentrations (4–250 μg/mL) (Figure 3c). In the present study, a concentration-dependent increase in inhibition was observed, reaching a maximum of 94.5% at 62.5 µg/mL, after which no further significant increase was detected at higher concentrations (Figure 3b,c). Swidan et al. reported that ginger-mediated AgNPs at 18 μg/mL reduced E. faecalis biofilm formation by 39.14% [72]. Similarly, Habibipour et al. found that AgNPs with sizes ranging from 32 to 85 nm significantly inhibited *P. aeruginosa* biofilm formation at concentrations of 100–500 μg/mL [73]. In another study, 50 μg/mL of AgNPs reduced *P. aeruginosa* biofilm formation by 86.73%. Mohanta et al. observed that AgNPs derived from *G. lanceolarium*, with an average size of 93.23 nm, required a concentration of 68.94 μg/mL to achieve 50% inhibition of biofilm formation [24]. Remarkably, AgNPs exhibited significant antibiofilm activity (94.5%) at a lower concentration (62.5 µg/mL) than reported for comparable materials, potentially reducing cytotoxicity and improving biocompatibility.

### 4.3. Molecular Docking

In the current study, molecular docking simulations were conducted to evaluate the antimicrobial and antibiofilm potential of silver oxide, complementing the in vitro analysis. *P. aeruginosa* is a common Gram-negative pathogen known to cause severe infections, particularly in individuals with cystic fibrosis or compromised immune systems. Additionally, certain strains of *P. aeruginosa* can infect both plants and animals [74]. *P. aeruginosa* enhances its survival by producing a variety of virulence factors, including exotoxins, pyocyanin, elastase, biofilms, and proteases. Additionally, it is well established that the quorum-sensing (QS) system plays a crucial role in regulating the expression of these virulence factors and in biofilm formation [75].

It has been reported that *P. aeruginosa* has three major quorum-sensing (QS) pathways: Las, Pqs, and Rhl [74]. Las is one of the quorum-sensing (QS) systems in *P. aeruginosa*, and LasR is a transcriptional activator that serves as a key regulator of the entire QS system [76]. Additionally, the *Pseudomonas* quinolone signaling (Pqs) system has emerged as a potential antibacterial target for combating *Pseudomonas aeruginosa* infections [75].

Furthermore, the secreted exopeptidase, *P. aeruginosa* aminopeptidase (PaAP; pepB, PA14_26020), is one of the most abundant proteins in the biofilm matrix and plays an important role in *P. aeruginosa* survival, including virulence and nutrient acquisition [77,78]. Thus, LasR, PaAP, and PqsA are considered important targets for the development of new inhibitors to treat cystic fibrosis and combat hospital-acquired infections, given their roles in *Pseudomonas aeruginosa* virulence. Molecular docking simulations were conducted to investigate the binding interactions between silver oxide and the protein targets PaAP, LasR, PqsA, and Pgd, which play key roles in microbial pathogenesis and biofilm formation.

Molecular docking studies have been conducted between Ag_2_O and different proteins. Jini et al. reported that, via molecular docking, silver molecules interacted with carbohydrate-hydrolyzing enzymes and insulin, potentially modulating their activity [79]. In another study, the Bcl-XL protein exhibited positive molecular binding interactions with silver nanoparticles derived from *Elaeagnus pyriformis*, with a binding energy of −6.5 kcal/mol [80]. Molecular docking analysis revealed interactions between the optimized Ag_3_ cluster and the *P. aeruginosa* protein, with binding energies ranging from −1.89 to −3.68 kcal/mol [81]. Additionally, in another study, molecular docking results indicated that AgCl-NPs showed a strong binding affinity of −10.30 kcal/mol toward the penicillin-binding proteins (PBPs) of *A. baumannii* [82]. Similarly, molecular docking simulations in the present study revealed binding affinities between the ligand and the Pgd, PaAP, LasR, and PqsA proteins of −3.84, −3.67, −4.25, and −5.63 kcal/mol, respectively (Figure 4a–d, Table 2). Although the calculated binding energies (−3 to −5 kcal/mol) showed relatively weak individual interactions, these values were typical for nanoparticle-protein binding studies. The interaction between the LasR protein and Ag_2_O ligand stabilized the complex through two hydrogen bonds with Leu40 and Tyr64 residues, as well as the formation of metal bonds with the Tyr40 residue (Table 2). Leu40 and Tyr64 were residues located in the active site of the LasR protein [74].

Four hydrogen bonds and one carbon-hydrogen bond were formed between the silver oxide and PaAP. Additionally, five metal-acceptor bonds contributed to the stabilization of the protein-ligand complex through hydrophobic interactions. The residues Tyr381, Met370, Glu418, Asp369, and Leu438, which form hydrogen bonds with ligands, were the same binding residues as those for the specific ligand of PaAP (Zn ion) [78].

The key residues contributing to the affinity of the PqsA–silver oxide complex included hydrogen bond-forming residues (Asn84, Tyr163, and Gly210) as well as residues involved in hydrophobic interactions, including carbon–hydrogen and metal–acceptor bonds (Table 2). Additionally, it could be inferred that the residues Asn84, Tyr163, and Gly210 in the active site of PqsA [75] enhanced the binding affinity of the silver oxide.

Peptidoglycan, which serves as one of the primary protective barriers in the bacterial cell wall, is composed of a peptide-linked heteropolymer of N-acetylglucosamine and N-acetylmuramic acid [83]. N-acetylglucosamine deacetylase Bc1974 from *B. cereus* plays a crucial role in virulence and resistance to host lysozyme. Thus, it is a key target of the innate immune system, and its degradation typically results in bacterial cell lysis [84]. The interaction between the Pgd protein and the silver oxide was analyzed through molecular docking. Two hydrogen bonds were formed in the Pgd-silver oxide complex through Asp80 and Thr227 residues. Also, the hydrophobic interaction between the silver oxide and the Pgd complex was stabilized through the Asp80 and Asp81 residues. Asp80 and Asp81 were key residues in the active site; additionally, Asp80 was the catalytic residue within it [85]. It should be noted that the reported hydrogen-bond and metal-acceptor interactions were predicted using a simplified Ag_2_O molecular model. Therefore, these interactions represent theoretical atom-level interactions and should not be interpreted as the actual binding behavior of the biosynthesized Ag/Ag_2_O nanoparticles, whose size, morphology, and phytochemical coating were not considered in the computational model.

Das et al. reported that gentamicin is an effective antimicrobial and antibiofilm agent against *Pseudomonas aeruginosa*. Therefore, gentamicin was used as the reference drug in the molecular docking analysis [86]. Moreover, gentamicin was used as the positive control in the antimicrobial assays performed in this study, enabling a consistent comparison between the experimental and molecular docking results. Quorum–sensing–related targets, including LasR and PqsA, showed stronger binding affinities than the reference drug gentamicin, supporting the antibiofilm activity at the molecular level, while gentamicin exhibited higher affinity for PaAP (Table 2). The substantially stronger predicted binding affinity of gentamicin toward PaAP compared with the Ag_2_O model may be attributed to the structural properties of gentamicin. As an aminoglycoside antibiotic, gentamicin possesses multiple amino and hydroxyl groups that may form numerous hydrogen bonds and electrostatic interactions within the PaAP binding pocket. In contrast, Ag_2_O provides fewer interaction sites, resulting in a less favorable predicted binding affinity.

The docking protocol was validated by redocking the co-crystallized ligand (N-3-oxo-dodecanoyl-L-homoserine lactone) into the LasR binding pocket using the same docking parameters. The redocked pose yielded an RMSD value of 1.607 Å relative to the crystallographic ligand, indicating successful reproduction of the experimental binding mode (Figure S1).

These docking findings correlated well with the in vitro results, which showed that AgNPs exhibited strong biofilm inhibition against *P. aeruginosa*. Therefore, the docking results provided in silico evidence at the molecular level supporting the proposed mechanisms underlying the biological efficacy of model silver oxide against target biofilm-related proteins.

### 4.4. Photocatalytic Activity

Several important factors influence photocatalytic degradation, including light absorption, contact time, pH, temperature, and others [87]. The spherical structure and particle size of silver nanoparticles also play a significant role in their photodegradation capability [21,88]. It is implied that AgNPs stabilized by phytochemicals can facilitate the attachment of dye molecules to their surface through van der Waals forces and hydrophobic interactions [54]. The photocatalytic performance observed in the present study was comparable to that of previously reported green-synthesized AgNPs. It was reported that 10 mg of AgNPs mediated by honey showed 20% degradation of 100 mL of 10 ppm MB in 2 h [88]. Similarly, Trieu et al. reported that 10 mg of AgNPs synthesized from Camellia sinensis extract showed a 69.5% degradation rate within 24 h [89]. In another study, 47% of 100 mL of 10 ppm MB was degraded by the same amount of AgNPs [90]. In this study, 1 mg of AgNPs rapidly and efficiently degraded 20 mL of 10 ppm MB within 3 h, showing consistency with previously published reports (Table 4).

It was reported that the Ag–CeO_2_ BMNPs at a dosage of 500 mg/L were photodegraded by 85% in a 25 mg/L EBT solution [91]. In the present study, AgNPs of 50 mg/L exhibited a maximum degradation efficiency of 29.5% against the EBT solution. The photocatalytic efficiency of silver nanoparticles for the degradation of various dye molecules has been well explored in the literature, but very limited reports have reported the photodegradation of EBT, an anionic dye. It was observed that AgNPs showed higher degradation efficiency against MB (60.6%) than EBT (29.5%) (Figure 5c). This difference may be associated with the distinct physicochemical properties of the dyes, including their molecular structure, charge, and adsorption behavior on the catalyst surface. Although previous studies have reported that solution pH can influence the degradation of cationic and anionic dyes [92], the effect of pH was not systematically investigated in the present study; therefore, no direct conclusion regarding its contribution can be drawn. Thus, positively charged cationic dyes were attracted to an alkaline intermediate, while negatively charged anionic dyes were drawn to an acidic intermediate due to their opposite charges [92]. In the present study, silver/silver oxide nanoparticles showed better activity towards the cationic dye.

Based on the available literature, the observed photocatalytic activity may be explained by the following proposed mechanism. Upon irradiation with sunlight, Ag_2_O/Ag nanoparticles absorb photons with energy equal to or greater than their bandgap, resulting in the excitation of electrons from the valence band (VB) to the conduction band (CB). This process generates electron–hole (e^−^/h^+^) pairs, leaving positively charged holes in the VB and free electrons in the CB. The photogenerated electrons subsequently react with dissolved oxygen molecules to produce superoxide radicals (•O_2_^−^), whereas the holes interact with water molecules or hydroxide ions to generate highly reactive hydroxyl radicals (•OH). In addition, other reactive oxygen species (ROS), such as hydrogen peroxide (H_2_O_2_) and hydroperoxyl radicals (•OOH), may also be formed during the photocatalytic process [39,40]. These ROS are highly oxidative and play a crucial role in degrading organic dyes by breaking their molecular structure into smaller intermediates and, ultimately, into harmless end products such as CO_2_ and H_2_O. Therefore, the photocatalytic degradation of MB/EBT may be attributed not only to dye adsorption on the nanoparticle surface but also to the generation of ROS facilitated by the Ag_2_O/Ag nanoparticles under sunlight irradiation.

**Table 4:** Comparison of the photocatalytic dye degradation performance of silver nanoparticles reported in previous studies.

Source of Nanoparticle Synthesis	Size (nm)	Dye(s)	Nanoparticle Concentration	Exposure Time	Photodegradation (%)	Reference
*Camellia sinensis*	25–40	Methylene blue	10 mg/L	72 h	95	[89]
Honey	5–25	Methylene blue	10 mg/100 mL	4 h	45	[88]
*Isoimperatorin*	79–200	Methylene blue	5 mg/25 mL	1 h	92	[6]
*Morinda tinctoria*	79–96	Methylene blue	10 mg/100 mL	72 h	95	[90]
Pure AgNPs	—	Eriochrome Black T	20 mg/50 mL	5 min	85	[93]
*Ruellia tuberosa*	55.65	Coomassie Brilliant Blue; Methylene blue	50 mg/100 mL; 10 mg/100 mL	3 h; 72 h	74; 87	[94]
*Sapindus mukorossi*	64	Eriochrome Black T	100 mg/100 mL	45 min	~90–98	[95]
*Streptomyces tuirus*	64	Methylene blue	5 mg/50 mL	6 h	71.3	[51]
*Valeriana jatamansi*	—	Rhodamine B	200 mg/100 mL	75 min	95	[4]
*Lotus sanguineus*	104.7	Methylene blue; Eriochrome Black T	1 mg/10 mL	180 min	60.6;29.5	Present study

Generally, there are several methods, such as adsorption and coagulation, to eliminate dyes from wastewater by converting them into a different phase. Those methods require additional processes, such as filtration and degradation, for full removal [20,96]. Photodegradation of organic dye waste is an efficient alternative due to its simple, economical, and non-toxic procedure. The use of nanomaterials under sunlight is emerging as one of the most effective green methods for removing organic dyes from contaminated water [6,96,97]. The results showed that AgNPs mediated by *Lotus sanguineus* at low concentrations might be a good alternative for removing organic dye waste, especially that generated in the textile industry, which is very harmful to the environment. Moreover, the moderate zeta potential value and the presence of phytochemical coating materials suggest reasonable colloidal stability, which may help maintain activity during repeated use. However, their environmental impact and scalability require further assessment, particularly regarding the recovery, ecotoxicity, and reusability of nanoparticles in real wastewater systems.

## 5. Conclusions

This was the first study to demonstrate the successful use of the endemic *Lotus sanguineus* extract to synthesize AgNPs in a simple, economical, and eco-friendly manner. AgNPs showed excellent photocatalytic activity against MB at very low concentrations. Therefore, under sunlight, AgNPs achieved degradation efficiencies of 60.6% for MB and 29.5% for EBT. It was also found that AgNPs had better photocatalytic activity towards a cationic dye than towards an anionic dye. AgNPs exhibited MIC values of 31.25–62.5 μg/mL and MBC values of 125–250 μg/mL against the tested bacterial strains. Furthermore, molecular docking results showed that the binding affinities between the ligand and Pgd, PaAP, LasR, and PqsA proteins were −3.84, −3.67, −4.25, and −5.63 kcal/mol, respectively. Accordingly, the molecular docking results provided computational support to in vitro analysis for the interactions underlying the biological activity of the synthesized nanoparticles toward biofilm-associated proteins. AgNPs might be a useful material for pharmaceutical applications due to their effective antimicrobial and bactericidal activity against tested bacteria, as well as promising biofilm-inhibition properties (94.5%) against *P. aeruginosa* ATCC 27853. The photocatalytic and antibiofilm activities of biosynthesized silver nanoparticles highlighted their potential for pharmaceutical applications, their suitability for integration into surfaces prone to bacterial colonization, and their promise as a solution to environmental pollution by embedding them in a membrane to improve dye degradation and dye salt separation. In future studies, the molecular mechanisms underlying the antimicrobial and antibiofilm activities of silver nanoparticles could be elucidated. Additionally, the development of pharmaceutical formulations or coating materials incorporating silver nanoparticles may be explored for potential biomedical applications. Further investigation is recommended to integrate biosynthesized silver nanoparticles into membrane systems to enhance antifouling and self-cleaning efficiency. In general, while the green synthesis approach and multifunctional assessment constitute the main strengths of this study, limitations, such as the lack of toxicity assessment and limited large-scale applicability, require further research.

## Figures and Tables

**Table 1:** Minimum inhibitory concentration (MIC) and minimum bactericidal concentration (MBC) values of the biosynthesized AgNPs against the tested bacterial strains.

Microorganisms	*E. coli*	*B. cereus*	*P. aeruginosa*	*S. aureus*
MIC (μg\mL)	62.5	62.5	31.25	62.5
MBC (μg\mL)	125	125	125	250

**Table 2:** The interaction of the silver oxide ligand with target proteins and the average binding affinity.

Proteins	Distance	Types	Residue in Contact	Affinity of Ag_2_O (kcal/mol)	Affinity of Gentamycin (kcal/mol)
Pgd	1.793	Hydrogen Bond	THR227	−3.84	−3.29
	2.741	Hydrogen Bond	ASP80		
	3.103	Metal-Acceptor	ASP81		
	3.166	Metal-Acceptor	ASP80		
PaAP	2.194	Hydrogen Bond	TYR381	−3.67	−9.46
	3.108	Hydrogen Bond	MET370		
	2.702	Hydrogen Bond	GLU418		
	2.854	Hydrogen Bond	ASP369		
	2.758	Hydrogen Bond	LEU438		
	3.473	Carbon Hydrogen Bond	ARG422		
	3.222	Metal-Acceptor	ASP369		
	2.833	Metal-Acceptor	TYR381		
LasR	1.972	Hydrogen Bond	LEU40	−4.25	−3.16
	2.223	Hydrogen Bond	TYR64		
	3.136	Metal-Acceptor	TYR47		
PqsA	2.084	Hydrogen Bond	ASN84	−5.63	−2.23
	2.112	Hydrogen Bond	TYR163		
	2.047	Hydrogen Bond	GLY210		
	3.097	Carbon Hydrogen Bond	GLN162		
	3.094	Metal-Acceptor	VAL82		
	2.703	Metal-Acceptor	THR164		
	2.742	Metal-Acceptor	TYR163		
	3.259	Metal-Acceptor	PHE208		

**Table 3:** Grid box center coordinates and dimensions used for molecular docking of the selected protein targets.

Protein	Grid Center (x, y, z)	Grid Size	Grid Spacing (Å)
LasR	(−40.179, 33.815, 24.989)	90 × 90 × 90	0.375
PqsA	(−9.476, 20.638, −13.794)	90 × 90 × 90	0.375
PaAP	(−14.407, 16.729, −40.515)	90 × 90 × 90	0.375
Pgd	(−13.838, −34.155, −15.584)	90 × 90 × 90	0.375

## Data Availability

All data generated or analyzed during this study are included in this published article.

## Supplementary Materials

The following supporting information can be downloaded at: https://www.mdpi.com/article/10.3390/biology15151261/s1, Figure S1: Biofilm formation of *P. aeruginosa* at different nanoparticle concentrations.; Figure S2: Docking validation by redocking the co-crystallized ligand (N-3-oxo-dodecanoyl-L-homoserine lactone) into the LasR binding pocket (PDB ID: 4NG2).
