# Supplementary material for: Biosynthesis of Silver Nanoparticles from Lotus sanguineus: Photocatalytic, Antimicrobial, and Antibiofilm Activities with Molecular Docking Against Quorum-Sensing Proteins
Source: Biology (Basel). 2026 Jul 31;15(15):1261. doi: 10.3390/biology15151261 (PMC13464980; doi:10.3390/biology15151261)
Supplement: Supplementary file 1 [file biology-15-01261-s001.zip › biology-4415959-supplementary.pdf]

Biosynthesis of Silver Nanoparticles from *Lotus sanguineus*: Photocatalytic, Antimicrobial, and Antibiofilm Activities with Molecular Docking Against Quorum-Sensing Proteins

Suzan Şahin Doğan

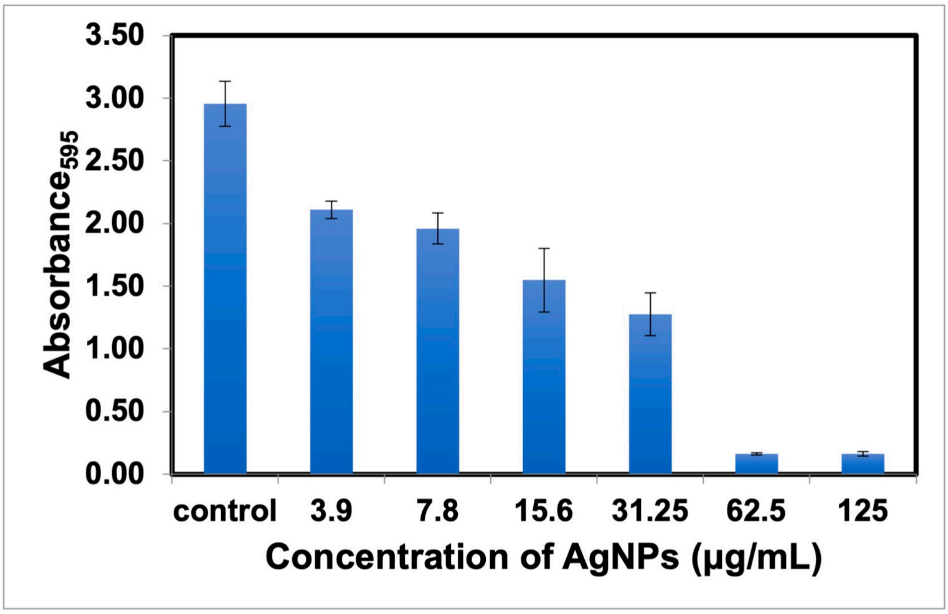

Figure S1. Biofilm formation of *P. aeruginosa* at different nanoparticle concentrations.

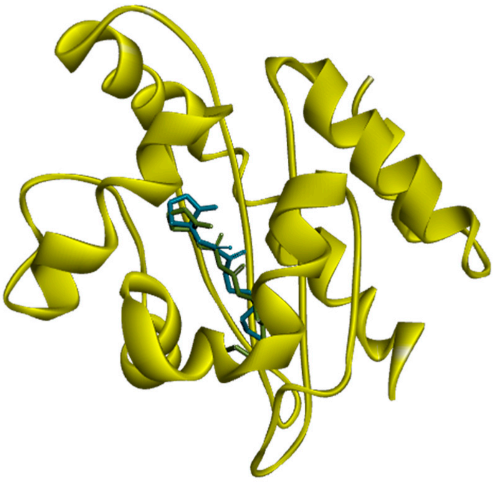

Figure S2. Docking validation by redocking the co-crystallized ligand (N-3-oxo-dodecanoyl-L-homoserine lactone) into the LasR binding pocket (PDB ID: 4NG2)
